# Computationally Designed Anti-LuxP DNA Aptamer Suppressed Flagellar Assembly- and Quorum Sensing-Related Gene Expression in *Vibrio parahaemolyticus*

**DOI:** 10.3390/biology11111600

**Published:** 2022-11-01

**Authors:** Nur Afiqah Md Yusof, Siti Aisyah Razali, Azyyati Mohd Padzil, Benjamin Yii Chung Lau, Syarul Nataqain Baharum, Nor Azlan Nor Muhammad, Nurul Hanun Ahmad Raston, Chou Min Chong, Natrah Fatin Mohd Ikhsan, Magdalena Lenny Situmorang, Low Chen Fei

**Affiliations:** 1 Institute of Systems Biology (INBIOSIS), Universiti Kebangsaan Malaysia, Bangi 43600, Selangor, Malaysia; 2 Faculty of Science and Marine Environment, Universiti Malaysia Terengganu, Kuala Nerus 21030, Terengganu, Malaysia; 3 Malaysia Genome and Vaccine Institute (MGVI), National Institute of Biotechnology Malaysia (NIBM), Jalan Bangi, Kajang 43000, Selangor, Malaysia; 4 Malaysian Palm Oil Board, Persiaran Institusi, Bandar Baru Bangi, Kajang 43000, Selangor, Malaysia; 5 Department of Biological Sciences and Biotechnology, Faculty of Science and Technology, Universiti Kebangsaan Malaysia, Bangi 43600, Selangor, Malaysia; 6 Department of Aquaculture, Faculty of Agriculture, Universiti Putra Malaysia, Serdang 43400, Selangor, Malaysia; 7 School of Life Sciences and Technology, Institut Teknologi Bandung, Bandung 40132, Indonesia

**Keywords:** single-stranded DNA aptamer, quorum sensing, LuxP, in silico modeling, molecular dynamics simulation, isothermal titration calorimetry, flagellar assembly, *Vibrio parahaemolyticus*

## Abstract

**Simple Summary:**

Infectious diseases are among the problems facing the global aquaculture industry, particularly Vibriosis, a bacterial infection caused by the *Vibrio* species. In shrimp farming, *Vibrio parahaemolyticus* causes the disease known as acute hepatopancreatic necrosis disease, which can lead to a 100% mortality rate of the infected shrimp. The use of antibiotics in aquaculture disease management is common; however, the misuse and overuse of antibiotics has led to the emergence of antibiotic resistance, demanding the development of alternative therapeutic agents for disease control and management. This study aimed to develop a potential therapeutic aptamer using a computational biology approach. The aptamer was designed to target the quorum-sensing receptor protein, which is involved in the regulation of bacterial pathogenicity. In this study, the aptamer exhibited anti-quorum-sensing properties and suppressed the flagellum gene expression. The flagellum is crucial in bacterial motility, and it is involved in the adhesion to, and invasion of, the host, causing infection. The findings of this study demonstrated the feasibility of developing the anti-quorum-sensing aptamer as a potential alternative therapeutic agent to control the *Vibrio* infection in the aquaculture industry.

**Abstract:**

(1) Background: Quorum sensing (QS) is the chemical communication between bacteria that sense chemical signals in the bacterial population to control phenotypic changes through the regulation of gene expression. The inhibition of QS has various potential applications, particularly in the prevention of bacterial infection. QS can be inhibited by targeting the LuxP, a periplasmic receptor protein that is involved in the sensing of the QS signaling molecule known as the autoinducer 2 (AI-2). The sensing of AI-2 by LuxP transduces the chemical information through the inner membrane sensor kinase LuxQ protein and activates the QS cascade. (2) Methods: An in silico approach was applied to design DNA aptamers against LuxP in this study. A method combining molecular docking and molecular dynamics simulations was used to select the oligonucleotides that bind to LuxP, which were then further characterized using isothermal titration calorimetry. Subsequently, the bioactivity of the selected aptamer was examined through comparative transcriptome analysis. (3) Results: Two aptamer candidates were identified from the ITC, which have the lowest dissociation constants (K_d_) of 0.2 and 0.5 micromolar. The aptamer with the lowest K_d_ demonstrated QS suppression and down-regulated the flagellar-assembly-related gene expression. (4) Conclusions: This study developed an in silico approach to design an aptamer that possesses anti-QS properties.

## 1. Introduction

*Vibrio parahaemolyticus* has been identified as the causative agent of early mortality syndrome, which is also known as acute hepatopancreatic necrosis disease (AHPND) in shrimp aquaculture. It is a Gram-negative, halophilic bacterium that thrives in warm climates within marine or estuarine environments [1,2]. In general, the species is one of the opportunistic pathogens harboring a large repertoire of virulence determinants that include thermostable direct hemolysin (TDH), TDH-related hemolysin (TRH), secretion systems, and more [1]. Meanwhile, the AHPND-causing *Vibrio parahaemolyticus* expresses a binary PirAB toxin that is encoded in a conjugative plasmid, which is responsible for the necrosis of the hepatopancreas in the infected shrimp. It is known to produce three types of autoinducers, namely the harveyi autoinducer 1 (HAI-1), autoinducer 2 (AI-2), and cholerae autoinducer 1 (CAI-1) [3,4], which are extracellular signaling molecules that mediate quorum sensing (QS). QS is a form of cell-density-dependent intercellular communication that enables the synchronization of bacterial behaviors at the population level. At a low cell density, where the autoinducer level is below the threshold, the autophosphorylation of the cognate autoinducer receptor shuttles phosphate to LuxO (QS response regulator protein) [5,6]. The phosphorylated LuxO, with σ54, activates the production of quorum regulatory RNA (Qrr). Qrr are small RNAs that promote the translation of aphA, which is the master QS regulatory protein at a low cell density [5]. At a high cell density, a high concentration of accumulated autoinducers is detected by the respective membrane-anchored receptor proteins, which causes the dephosphorylation of downstream proteins and, subsequently, leads to the activation of OpaR (QS regulator at a high cell density) [4,7]. It has been demonstrated that QS, in *V. parahaemolyticus*, is also responsible for the regulation of virulence genes, such as the TDH and type-3 secretion system [8,9,10]. The critical role of QS in the virulence expression in *Vibrio* species has become more prominent than it was in the past. For instance, full virulence expression in *V. campbelli* required the activation of QS cascade by HAI-1 and AI-2, as essential signals, but not CAI-1 during the experimental infection of grouper larvae [11]. On the other hand, AI-2 and CAI-1 appeared to be dominant signals that were required by *V. harveyi* during the infection of brine shrimp [12]. In light of the importance of QS in the pathogenesis of *Vibrios*, the obstruction of the quorum sensing cascade appears to be a feasible therapeutic alternative. LuxP, a periplasmic protein, was found to be involved in the binding of AI-2, which then interacts with the inner membrane sensor kinase LuxQ to form a LuxPQ complex that transduces the AI-2 information to cytoplasm through the QS cascade [13,14]. This LuxP receptor protein appeared to be a potential target of quorum quenching strategies designed to obstruct the signal transduction of AI-2.

The current study aimed to model aptamers to be used against the LuxP receptor protein so as to interfere with the sensing of AI-2. Additionally, known as chemical antibodies, aptamers are single-stranded oligonucleotides (DNA or RNA) that can bind to a specific target with a high affinity and specificity, which is attributable to their unique three-dimensional structure in solution. The high binding affinity and specificity are comparable to, or even higher than, those of antibody–antigen bindings [15,16]. The aptamers have benefits in clinical applications that include immunophenotyping and the use of biosensors for disease detection, such as tumor or cancer cell markers, etc. [17,18,19]. Numerous therapeutic aptamers that are in preclinical and clinical development have been reported, but the well-known therapeutic aptamer that has received US Food and Drug Administration (FDA) approval was the Macugen aptamer, which is used to treat the macular degeneration of the eye [20]. In addition, aptamer research in agriculture also revealed the potential of therapeutic aptamers as antiviral agents, which demonstrated antiviral properties against several important aquaculture pathogens, such as viral hemorrhagic septicemia virus, grouper nervous necrosis virus, and Singapore grouper iridovirus [21,22,23]. The aptamers are developed through SELEX (systematic evolution of ligands by exponential enrichment), which is an in vitro process that consists of iterative cycles of the selection and amplification of the oligonucleotides that bind to specific targets [19,24,25]. However, the in silico design and development of aptamers have emerged to complement SELEX in aptamer research. One of the prominent advantages of using a molecular modeling approach for aptamer design and development is the identification of the structural patterns that are essential for aptamer–target binding [26,27]. The computational-aided design and development of aptamers often involve docking and molecular dynamics simulation. The rigid or flexible docking of the aptamer and target can be performed to select aptamer–target complexes with the lowest binding energies. Molecular docking analysis is fundamental for predicting ligand–target interactions at the molecular level, which, in this case, are the aptamer–protein interactions. It has been widely used in virtual screening for drug design and drug repositioning and is increasingly reported with respect to its use in the selection and design of DNA/RNA aptamers against various target proteins [27,28,29,30]. The stability of the docked complex and the dynamics of the aptamer–target interaction could be further evaluated using molecular dynamic (MD) simulations. In addition, a higher accuracy of the calculation of the binding energies could be obtained from MD simulation compared to the previous rigid or flexible docking [31].

In this study, molecular docking was applied to select oligonucleotides with the lowest binding energy against the LuxP receptor protein, followed by the molecular dynamics simulation of the selected complexes. Subsequently, the binding thermodynamics of the selected aptamer candidates against the recombinant LuxP protein were analyzed using isothermal titration calorimetry. Lastly, the in vitro bioactivity of the selected aptamer was examined, and comparative transcriptome analysis was conducted to reveal the changes in pathogenicity- and quorum-sensing-related gene expression upon the aptamer treatments.

## 2. Materials and Methods

### 2.1. Molecular Docking and Dynamics Simulation

Due to the unavailability of a high-resolution crystal structure of the *Vibrio parahaemolyticus* LuxP receptor protein, the crystal structure of *V. harveyi* LuxP (apo form) complexed with LuxQ (PDB ID: 1ZHH) was downloaded from the RCSB Protein Databank, https://www.rcsb.org/ (accessed on 1 January 2020) and used for the molecular docking and dynamics simulation. The protein model was prepared by removing the LuxQ protein chain, water molecules, and the 2-[N-cyclohexylamino]ethane sulfonic acid molecule. Missing hydrogen atoms were added to the LuxP protein using the AutoDockTools-1.5.6. Prior to the molecular docking, the structure was energy-minimized using Chem3D 15.1 to achieve the minimum the RMS gradient of 0.01, and then the energy-minimized structure was converted into PDBQT format. A grid box sized 50 × 50 × 60 A3 was generated to contain the LuxP protein, where the binding pocket was set as a centroid. The molecular docking was performed using AutoDock Vina 1.1.2 38. The complex was further analyzed using UCSF Chimera version 1.15 to visualize the interactions between the proteins and oligonucleotides that were mediated by hydrogen bonds. A reference model of the LuxP-AI-2 (autoinducer 2) complex (PDB ID: 1JX6) was used to identify the key amino acids involved in the protein–ligand interactions. An initial library of 4-mer oligonucleotides was used to perform the molecular docking against the LuxP receptor protein. This consisted of 256 oligonucleotide candidates, where each position of the 4-mer oligonucleotides had all 4 possible combinations of the guanine, cytosine, adenine, and thymine nucleotides. The library was constructed using PerkinElmer ChemDraw Professional 15.1. The 4-mer oligonucleotides were then subjected to energy minimization using Chem3D 15.1 prior to the molecular docking. The energy-minimized structures were then converted into PDBQT format to proceed with the molecular docking analysis. Six 4-mer oligonucleotide candidates with a binding energy of ≤ 11 kcal/mol were selected. These candidates were then extended at the 3′ and 5′ ends by combination to obtain 8-mer oligonucleotides. Subsequently, the oligonucleotides of 8 mers with the highest affinity were selected (unpublished data) for the dynamics simulation using GROMACS 2019.6. The topology of the protein and 8-mer oligonucleotides were generated using the GROMOS 54A7 force field and PRODRG server, http://davapc1.bioch.dundee.ac.uk/cgi-bin/prodrg (accessed on 1 June 2020). The oligonucleotide–LuxP complexes were solvated in the center of the cubic box with a default 3-point SPC water model. The electrostatic energy was calculated and the system was neutralized by adding in counterions to create a neutralized system charge. Then, the subsequent energy minimization was performed using the steepest descent minimization of 50,000 steps. The system was further equilibrated for 100 ps at a constant volume and temperature of 300 Kelvin in sequence in two equilibration steps under an isothermal–isochoric (NVT) ensemble and then under an isothermal–isobaric (NPT) ensemble. The molecular dynamics simulation was run for 50,000 ps for each of the oligonucleotide–LuxP complexes, where the coordinates were recorded at every two fs interval. All the bonds were constrained using the LINear Constraint Solver (LINCS) algorithm during the 50,000 ps dynamics simulation, and the electrostatic interactions were treated with the adoption of the particle mesh Ewald method [32]. The MD simulations were analyzed using Grace [33] and Visual Molecular Dynamics [34].

Binding free energy calculations: The interaction energy of the oligonucleotide–LuxP complexes was calculated using the molecular mechanics Poisson–Boltzmann surface area (MMPBSA) approach [35]. The binding free energy of each complex was calculated from 20 snapshots at time intervals of 500 ps from 30,000 to 40,000 ps of the simulation. The components of the binding free energy estimation include the vacuum potential energy (bonded and non-bonded interactions) and the free energy of the solvation (polar and nonpolar terms), where the solvent accessible surface area (SASA) method was used to calculate the nonpolar solvation energy term [36].

### 2.2. Production and Purification of the Recombinant LuxP Receptor Protein

The sequence of the *V. parahaemolyticus* LuxP gene (VP_RS21025) was retrieved from the National Center for Biotechnology Information (NCBI), https://www.ncbi.nlm.nih.gov/ (accessed on 1 August 2020). The encoding region was synthesized and cloned into vector pET28b(+) flanking the NcoI and XhoI recognition site, adjacent to the 6x His-tag gene sequence. The recombinant plasmid was then transformed into *E. coli* for the recombinant LuxP protein expression. The transformant was cultured in 10 mL LB broth supplemented with Kanamycin at 37 °C with constant shaking at 200 rpm, and then upscale-cultured in 1 L LB broth with 25 µg/mL Kanamycin supplementation until the OD_600_ achieved 0.6. Isopropyl β-d-1-thiogalactopyranoside (IPTG) at an amount of 1 mM was then added to induce the protein expression by incubation at 25 °C for 4 h. The cells were collected by centrifugation at 10,000× *g* at 4 °C for 5 min. The collected cells were stored in −20 °C prior to protein extraction. The recombinant protein was extracted using 6xHistidine-tag with modifications. The cells were lysed in 20 mL Ni—NTA lysis buffer (50 mM NaH_2_PO_4_, 300 mM NaCl and 10 mM imidazole, pH 8). A total of1 mg/mL lysozyme was added, and the sample was incubated on ice for 30 min. The sample was then sonicated using 10 s bursts and a 10 s cooling period for 5 min. A total of 5 µg/mL DNase was then added to the sample and incubated on ice for 15 min. The lysate was centrifuged at 10, 000× *g* at 4 °C for 10 min to pellet the cellular debris. In order to purify the recombinant LuxP protein, a HisTrap HP column (GE Healthcare, Chicago, IL, USA) was used, and the purification was carried out according to the manufacturer’s protocol, with modification. The extracted total protein was filtered using a sterilized 0.22 µm nitrocellulose filter. The column was washed with 25 mL degassed distilled water and equilibrated with washing buffer (50 mM NaH_2_PO_4_, 300 mM NaCl and 55 mM imidazole, pH 8) before the sample was applied to the column. Then, the column was washed with 65 mL of washing buffer. The purified LuxP protein was eluted in five elution fractions of 1 mL/fraction in the elution buffer (50 mM NaH_2_PO_4_, 300 mM NaCl and 250 mM imidazole, pH 8). The purified LuxP protein across all the elution fractions was documented using 1D SDS—PAGE, and the protein concentration was determined using a Bradford assay.

### 2.3. Protein Identification

Gel plugs containing the purified protein were de-stained with 0.2 M ammonium bicarbonate and reduced with 100 mM TCEP for 1 h at 60 °C. Alkylation was performed using 200 mM iodoacetamide for 45 min. Then, 1% (*w*/*v*) of sodium deoxycholate was added to the gel plugs, and they were incubated for 10 min at 37 °C. Tryptic digestion was performed with 0.1 µg trypsin for 16 h at 37 °C. Peptides were extracted from the gel plugs by adding 10% ACN in 5 mM ammonium bicarbonate, 50% ACN in 0.1% formic acid, and 80% ACN for the final elution. After extracting the peptides, 0.5% formic acid was added to precipitate out the sodium deoxycholate by incubating the mixture at 37 °C for 45 min, followed by centrifugation at 14,000 rpm for 15 min. The digest was then vacuum-dried. The peptides were reconstituted in 20 µL of 0.1% FA and 5% ACN. Digests of 2 µL were loaded onto an Acclaim PepMap 100 C18 column (2 µm, 0.075 × 150 mm) (Thermo Scientific, Waltham, MA, USA). The reverse-phase column was equilibrated with 0.1% FA (mobile phase A) and 80% of ACN containing 0.1% FA (mobile phase B). A gradient of 5–35% mobile phase B, at a flow rate of 300 nL/min, was applied for 75 min for the peptide elution. The separation of the peptides was accomplished using an EASY-nano liquid chromatography 1200 System (Thermo Scientific, Waltham, MA, USA). An online Q Exactive Plus Hybrid Quadrupole-Orbitrap mass spectrometer system (Thermo Scientific, Waltham, MA, USA) was used to generate the peptide ions with a spray voltage of 1900 V in the positive mode. A precursor ion scan was conducted with a resolution of 70,000 and a mass range of m/z 310–1800. The precursors exhibiting a charge state ranging from 2+ to 8+ were fragmented further. The fragmentation was performed via collision-induced and high-energy collision-induced dissociation (CID and HECID) at a normalized energy of 28%, correspondingly. The resolution, isolation window, and ion injection time were set to 17,500, 0.7 Da, and 60 ms, respectively. The scanned precursor mass range was set to m/z 110–1800. The mass spectra of the peptides were acquired using Tune (Ver. 2.11 QF1 Build 3006) (Thermo Scientific, Waltham, MA, USA) and deconvoluted using Proteome Discoverer (Ver. 2.4) (Thermo Scientific, Waltham, MA, USA) to create the peptide mass list. The SEQUEST HT search engine, incorporated into Proteome Discoverer, was used to match the generated mass list against the *V. parahaemolyticus* FASTA sequences downloaded from Uniprot. The mass tolerance of the peptides and their fragments was fixed at 20 ppm and 0.5 Da, respectively. Trypsin was selected as the digestion enzyme used, and up to two miscleavages were allowed during the search. The carbamidomethylation modification of cysteine residues was set as a static modification, while the variable amino acid modifications included deamidation (asparagine and glutamine residues) and oxidation (methionine residues). The mass list was also searched against a decoy database generated from the randomized protein sequences. The identified proteins had to have, at least, a Rank 1 peptide and a false discovery rate of 1% in order to be accepted. The spectra that matched the sequences were further validated with Percolator algorithm (Ver. 2.04) using the q-value at the 1% false discovery rate.

### 2.4. Isothermal Titration Calorimetry

A nano isothermal titration calorimeter (TA Instruments) was used to record the energy changes associated with the aptamer–LuxP interaction at 298 K. Both the aptamer and LuxP protein samples were degassed at 383 mmHg for 10 min prior to analysis. The purified LuxP protein (10 µM) and aptamers (100 µM) were loaded into the sample cell and injection syringe, respectively, of the calorimeter. The aptamer sample was titrated into the sample cell using 20 injections of 2.5 µL per injection at 200 s intervals to enable the equilibration and baseline stabilization post-injection. The sample cell was stirred throughout the process at 250 rpm. The heat changes in the sample cell corresponding to each injection were recorded, which were then transformed to obtain a plot of the enthalpy against molar ratio of the ligand:protein. The titration of the aptamers into protein buffer was used as the control. The thermodynamic parameters (enthalpy and entropy changes) and the dissociation constant, K_d_, were determined according to the best-fit model using the NanoAnalyze Data Analysis software, version 3.11.0, by TA Instruments.

### 2.5. Comparative Transcriptome Analysis of V. parahaemolyticus Treated with the Aptamer Candidate

In brief, the overnight culture of *V. parahaemolyticus* BpShHep31 in marine broth was conducted, and the OD_600_ was adjusted to 0.6. The culture broth was then replaced with fresh marine broth. The assay was conducted in triplicate in 3 mL of each culture volume, and the treatment groups consisted of a positive control, (5*Z*)-4-bromo-5-(bromomethylene)-2(5*H*)-furanone at a 10 µM treatment concentration, two aptamer treatment concentrations at 10 µM and 100 µM, and a negative control treated with PBS. All samples were incubated for 3 h at 30 °C and shaken at 200 rpm.

A culture volume of 1 mL from each replicate was obtained for the total RNA extraction. Bacterial cells were collected by centrifugation at 10,000 rpm for 5 min at 4 °C. The total RNA was extracted using the Monarch^®^ Total RNA Miniprep Kit (New England Biolabs, Hitchin, England) according to the manufacturer’s protocol. The extracted RNA was eluted in nuclease-free water and stored at −80 °C prior to further analysis. All the RNA samples passed the quality check, where an RIN number above 9.0 was recorded prior to the RNA sequencing. The RNA samples were then outsourced for the library preparation and subsequent sequencing by a local service provider. In brief, to construct the library for the sequencing, the RNA samples were first subjected to rRNA depletion using the NEBNext rRNA depletion kit (bacteria) prior to RNA fragmentation. Then, the fragmented RNA was reverse-transcribed to cDNA followed by second-strand synthesis, end-repair and A-tailing, adapter ligation, and PCR amplification. The amplified library was then subjected to a denaturation step, circularization, and digestion for subsequent sequencing using the DNBSEQ^TM^ sequencers for high-throughput sequencing.

Subsequently, the raw sequence data were filtered to remove low-quality reads and adapter contamination. The data filtering process included the removal of reads containing adapters, reads containing an unresolvable base ratio that was more than 5%, and reads containing >50% of low-quality (Qphred < 5) base ratios. Clean reads that achieved a Phred score of 20 or above were used for the subsequent analysis. The clean reads were mapped to the *Vibrio parahaemolyticus* RIMD 2210633 reference genome using Bowtie2 [37]. The latest version of the gene annotation file was then downloaded from the Refseq database to serve as a reference. The aligned RNA-seq reads were then quantified using featureCounts V2.0.1 [38]. Following this, DESeq2 [39] was adopted to perform the differential gene expression (DEG) analysis. In order to compare the gene expression levels between samples, data normalization was performed using DESeq2, where the geometric mean was calculated for each gene across all the samples. Genes that had zero or less than 10 mean read counts were omitted in the downstream analysis. Lastly, DESeq2-fitted negative binomial generalized linear models for each gene and Wald’s test were used for the significance testing. DEGs with a *p* adjusted value < 0.05 and log fold changes of >1 and <−1 were considered significant.

## 3. Results

### 3.1. Molecular Docking

The six top-ranked eight-mer oligonucleotides with the lowest binding energy were shortlisted (Table 1) based on the molecular docking analysis against the LuxP protein, where the predicted binding affinity ranged from −7.5 to −9.2 kcal/mol. The analysis of the best-docked position of the eight-mer oligonucleotides with the LuxP protein revealed that the VPL130 has the highest number of interacting amino acid residues, where 17 amino acid residues of the LuxP interacted with VPL130 through hydrogen bonds (Table 1).

**Figure 1:**
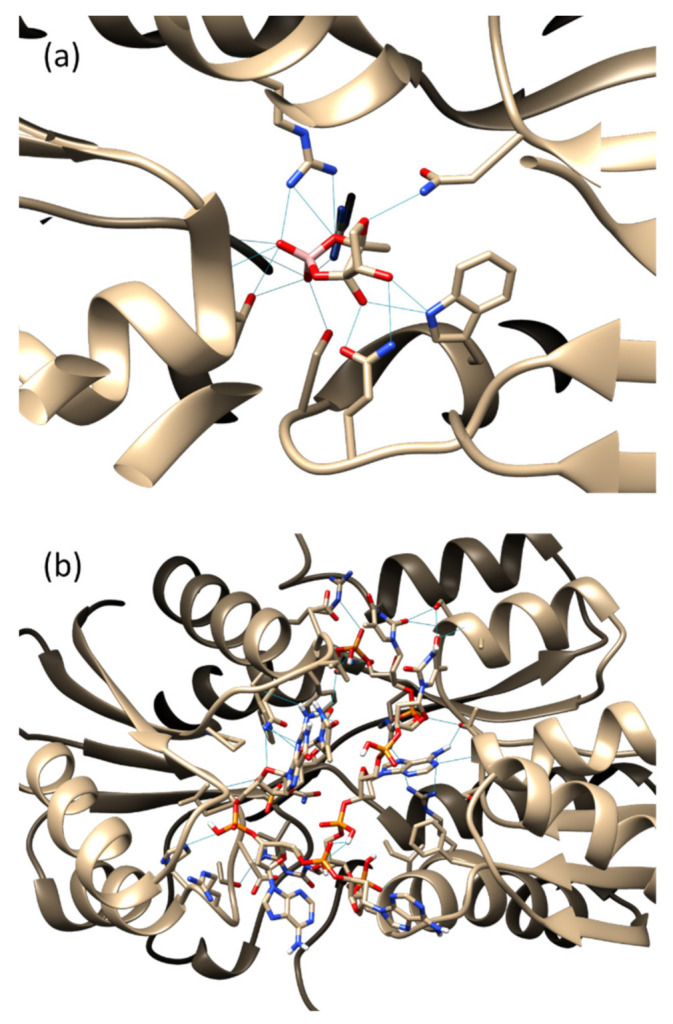
Protein–ligand complex interactions revealed hydrogen bonding between (**a**) AI-2, the QS signaling molecule (reference model of LuxP-AI-2 complex; PDB ID: 1JX6); (**b**) VPL130, with the LuxP receptor protein. Hydrogen bonds are presented as light blue lines. The graphical presentation was visualized using UCSF Chimera version 1.15.

In order to visualize the AI-2-binding domain of the LuxP receptor protein, a reference model of the LuxP–AI-2 complex (1JX6) was analyzed using UCSF Chimera version 1.15. The analysis revealed seven key amino acid residues, Ser79, Arg310, Arg215, Thr266, Asn159, Trp82, and Gln77, that interact with AI-2 through hydrogen bonds (Figure 1a).

**Figure 2:**
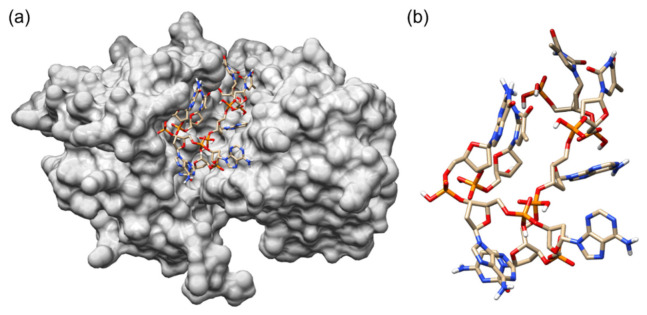
(**a**) Best-docked position of the 8 mer oligonucleotides (VPL130) in the binding pocket of the LuxP periplasmic receptor protein (protein shown on grey-colored surface) and (**b**) the docked structure of VPL130. The graphical presentation was visualized using UCSF Chimera version 1.15.

These key amino acid residues were also observed in the binding of the oligonucleotides (Table 1). Six out of the seven amino acids were found to be involved in the binding of VPL130, in addition to other 10 amino acid residues (Figure 1b, Table 1). The structure of the VPL130 that was docked in the LuxP binding pocket is presented in Figure 2.

### 3.2. Molecular Dynamics Simulation

**Figure 3:**
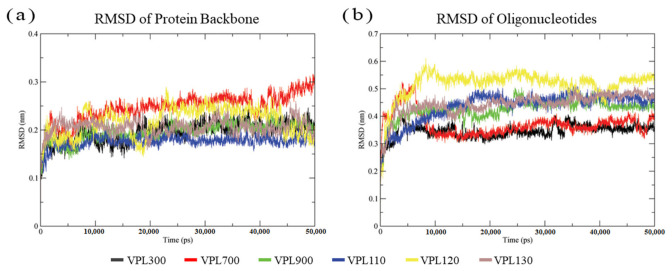
RMSD (nm) of the LuxP protein backbone (**a**) and oligonucleotides (**b**) over 50 ns of dynamics simulation.

The dynamics of the interactions were further examined through molecular dynamics simulation using GROMACS version 2019.6. The root mean square deviations (RMSD) of the protein backbone and oligonucleotides were recorded to measure the deviation of the protein and oligonucleotides’ structure conformation during the course of the simulation. From the 50 ns simulation, all six of the complexes presented with a stable protein backbone fluctuation that ranged between 1.5 and 2.5 Å, approximately (Figure 3a). However, a higher range of the oligonucleotides’ structural fluctuation was recorded, ranging between 3 and 5 Å (Figure 3b).

**Figure 4:**
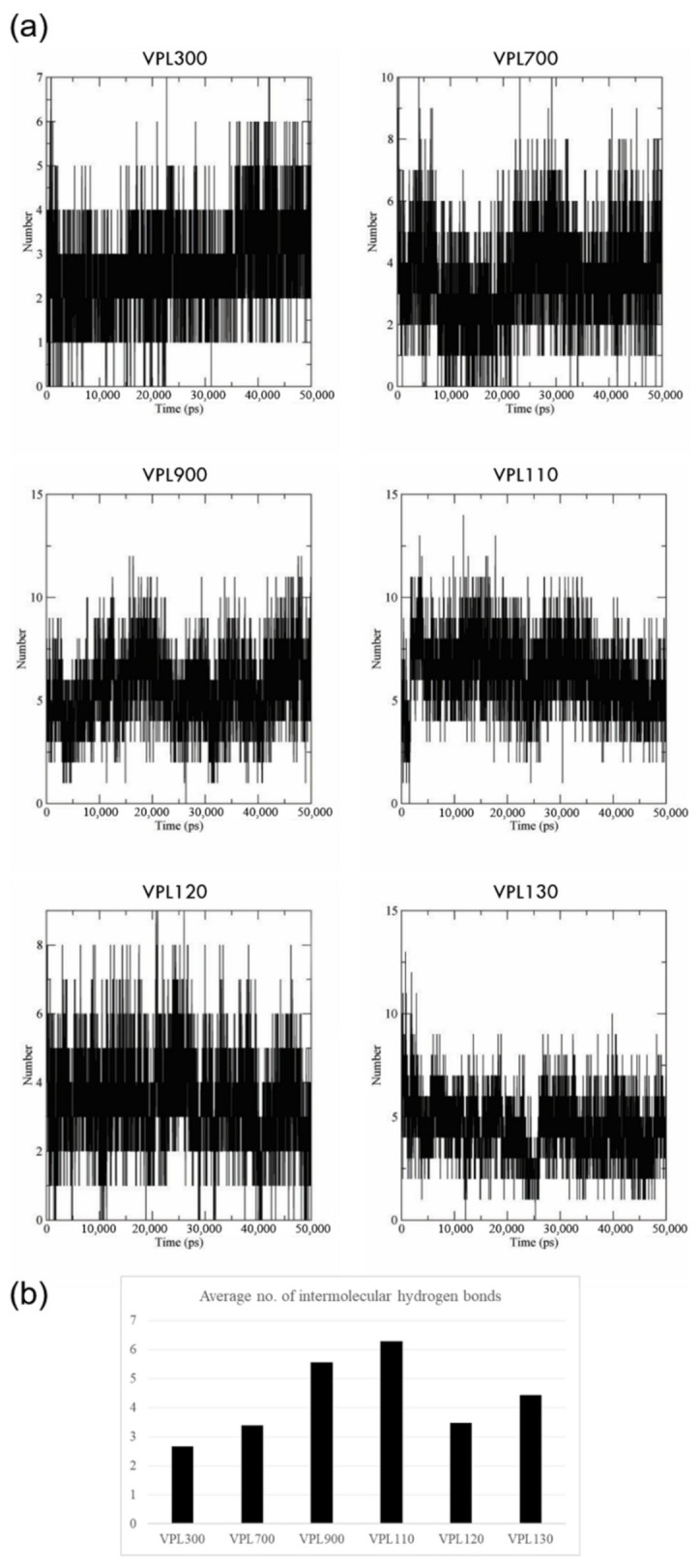
Intermolecular hydrogen bonds between the LuxP receptor protein and oligonucleotide for each complex during the course of the 50 ns MD simulation: (**a**) number of hydrogen bonds formed over 50 ns simulation; (**b**) average number of hydrogen bonds per timeframe.

The prevalence of intermolecular hydrogen bonds during 50 ns of MD simulation was assessed. All six of the oligonucleotide candidates showed stable binding based on the strong hydrogen bonding profile throughout the simulation (Figure 4a), where VPL110, VPL900, and VPL130 were recorded with the highest average numbers of hydrogen bonds, amounting to 6.3, 5.6, and 4.4 hydrogen bonds per timeframe, respectively (Figure 4b).

Nevertheless, the binding energy computed over the course of the MD simulation using the MMPBSA approach showed that VPL900 has the lowest binding energy of −326.981 ± 38.291 kJ/mol, followed by VPL120 and VPL130, with binding energies of −301.524 ± 24.673 kJ/mol and −293.980 ± 25.769 kJ/mol, respectively (Table 2).

### 3.3. Isothermal Titration Calorimetry

**Figure 5:**
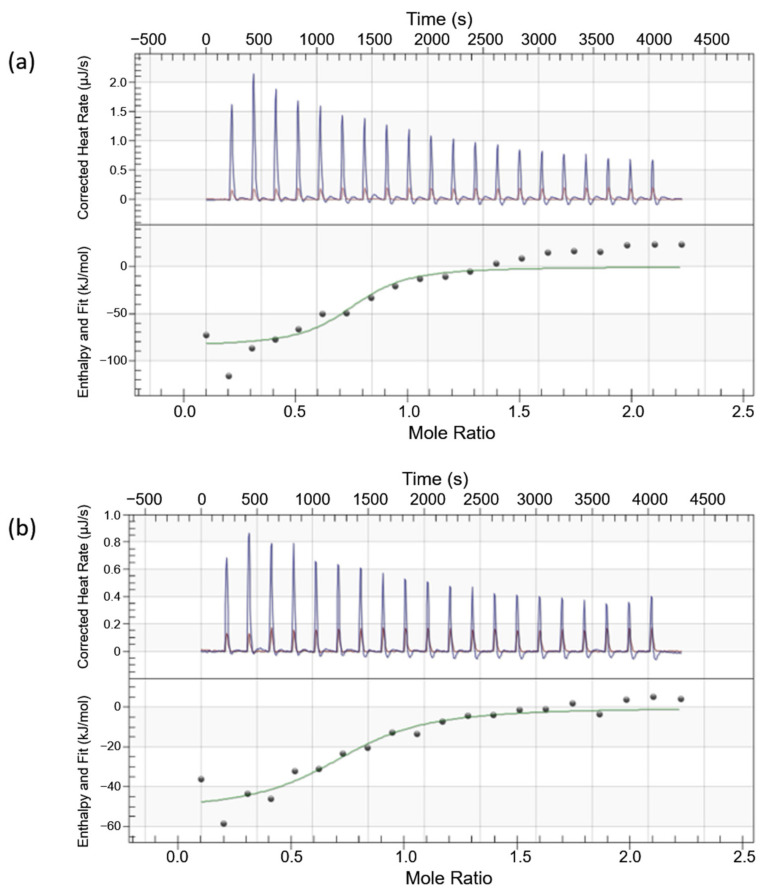
Titration profiles at 25 °C of (**a**) aptamer VPL300 and (**b**) aptamer VPL130 into LuxP. The titration of the aptamer into protein buffer was used as the control for heat rate correction.

The binding and interactions of the selected oligonucleotides with LuxP were then analyzed using isothermal titration calorimetry (ITC) in order to analyze the binding energies of the molecules. Prior to ITC analysis, the recombinant LuxP protein of *V. parahaemolyticus* was produced by cloning the LuxP gene into pET-28(b)+ vector, which was then transformed into *Escherichia coli* as the expression host. The recombinant LuxP (approximately 41 kDa) was purified (Figure S1), and the protein identity was confirmed through peptide sequencing (Table S1, Figure S2). On the other hand, the selected oligonucleotides (eight mers of single-stranded oligonucleotides in the linear form) were modified by adding self-complement sequences at the 5′ and 3′ ends in order to form a hairpin structure, which is relatively more stable than the linear strand of eight mers. The full sequence of the synthesized oligonucleotides was 5′-GGCC-(selected eight-mer oligos)-GGCC-3′. The interactions between the recombinant LuxP and modified oligonucleotides (hereafter referred to as aptamers) were investigated using ITC at 298 K. The aptamer was injected into sample cell of ITC containing the purified recombinant LuxP, which generated heat burst peaks that corresponded to each single injection (Figure 5).

Out of the six aptamer candidates, VPL300 and VPL130 showed a significant binding affinity, with dissociation constants K_d_ of 2.11 × 10^−7^ M and 5.64 × 10^−7^ M (Table 3), respectively. The thermodynamic parameters of the interactions are summarized in Table 3. VPL300 appeared to be the best candidate, with the lowest K_d_ and stoichiometry value closest to 1, indicating a single-site binding of VPL300 to the LuxP receptor protein.

Subsequently, VPL300 was selected and further analyzed for its bioactivities against *V. parahaemolyticus*. The bacterial culture was treated with low (10 µM) and high (100 µM) doses of VPL300, and the transcriptomes were sequenced to examine the effect of the aptamer treatment on the bacterial gene expressions. A bacterial culture treated with (5*Z*)-4-bromo-5-(bromomethylene)-2(5*H*)-furanone was included as a positive control, as this halogenated furanone is known for its anti-quorum-sensing properties.

### 3.4. Comparative Transcriptome Analysis

**Figure 6:**
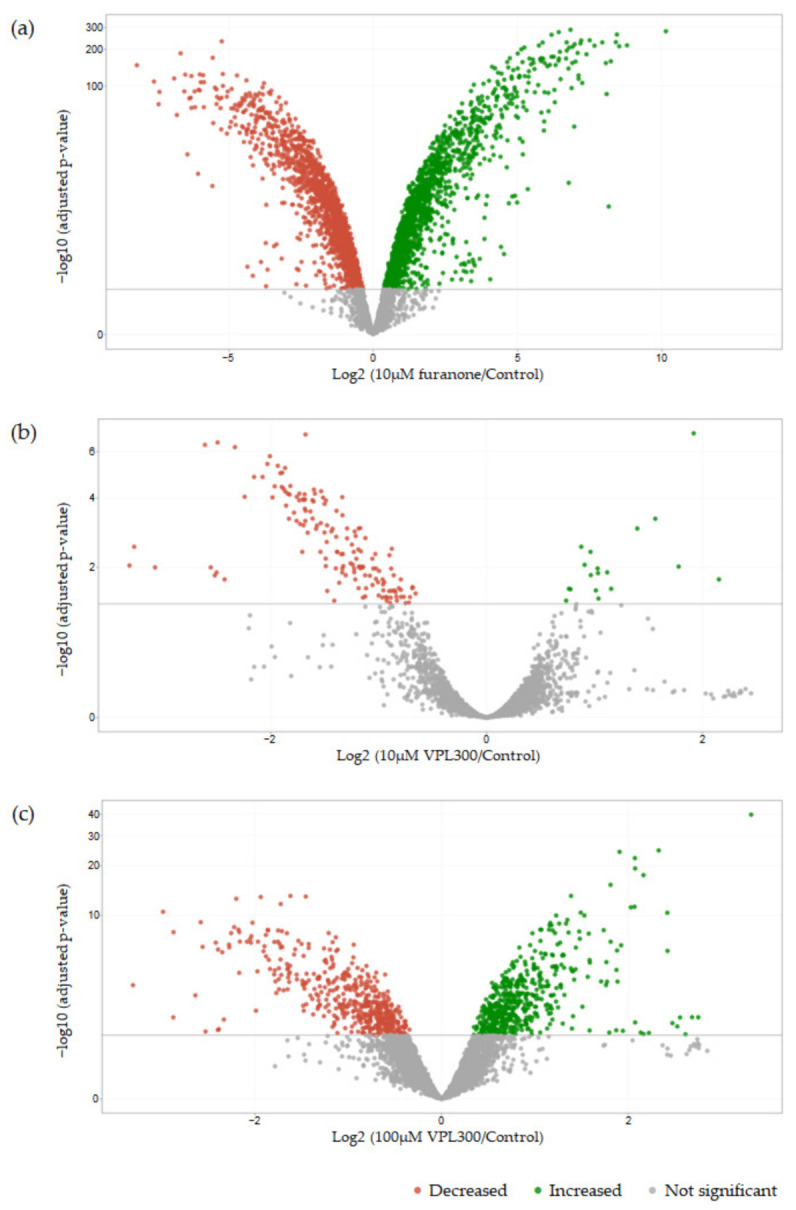
Volcano plot of differentially expressed genes. Plot of log-adjusted *p*-value against log_2_ fold change for (**a**) 10 µM furanone treatment/control; (**b**) 10 µM VPL300 treatment/control; and (**c**) 100 µM VPL300 treatment/control. Red- and green-colored dots represent the respective down- and up-regulated genes that were significant.

A pairwise comparison of the gene expression analysis revealed 3119 differentially expressed genes (DEGs) in *V. parahaemolyticus* treated with 10 µM of furanone compared to the control. Meanwhile, 165 DEGs and 1017 DEGs were identified in *V. parahaemolyticus* treated with 10 µM and 100 µM of VPL300, respectively, compared to the control. The DEGs for each pairwise comparison were summarized in Figure 6.

**Figure 7:**
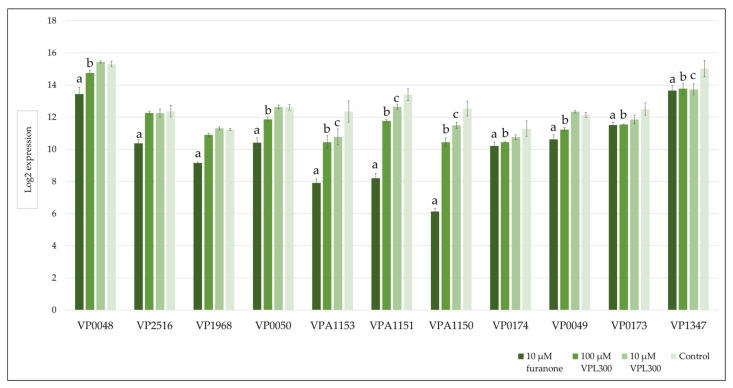
Expression levels of quorum-sensing-related genes in response to the furanone and VPL300 aptamer treatments compared to the control. a,b,c: significant difference in the expression level compared to the control; VP0048: peptide ABC transporter, periplasmic-peptide-binding protein; VP2516: OpaR; VP1968: sensor protein LuxN; VP0050: peptide ABC transporter, permease protein; VPA1153: putative high-affinity branched-chain amino acid transport ATP-binding protein; VPA1151: putative ABC transporter, membrane spanning protein; VPA1150: putative high-affinity branched-chain amino acid transport permease protein; VP0174: oligopeptide ABC transporter, ATP-binding protein; VP0049: peptide ABC transporter, permease protein; VP0173: oligopeptide ABC transporter, ATP-binding protein; VP1347: oligopeptide ABC transporter, periplasmic-oligopeptide-binding protein.

**Figure 8:**
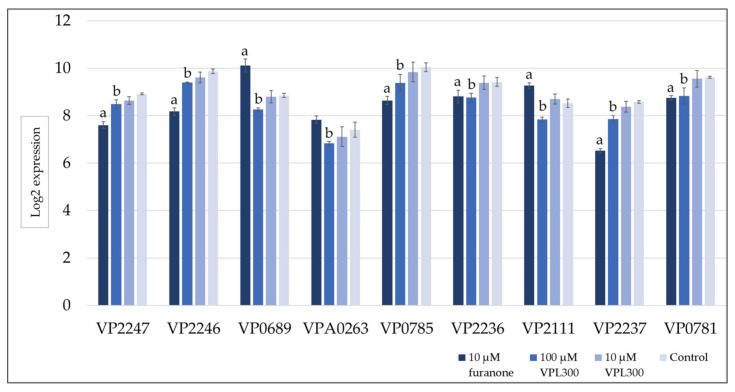
Expression levels of flagellar assembly-related genes in response to the furanone and VPL300 aptamer treatments compared to the control. a,b: significant difference in the expression level compared to the control; VP2247: polar flagellar assembly protein FliH; VP2246: polar flagellum-specific ATP synthase FliI; VP0689: sodium-driven polar flagellar protein MotA; VPA0263: LfgA; VP0785: polar-flagellar-hook-associated protein type 1 FlgK; VP2236: polar flagellar assembly protein FlhB; VP2111: sodium-type flagellar protein MotY; VP2237: polar flagellar assembly protein FliR; VP0781: polar flagellar FlgG.

**Figure A1:**
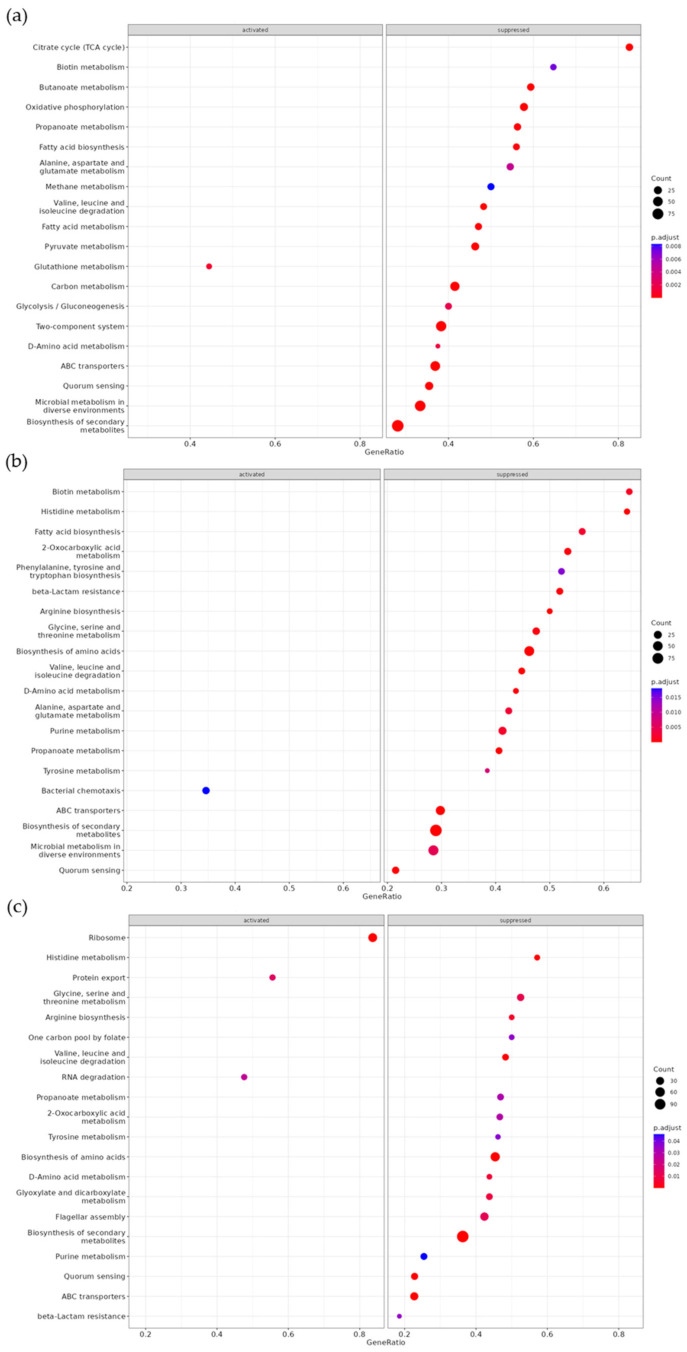
Gene set enrichment analysis showing the top 20 KEGG categories annotated with DEGs. Pairwise comparison between samples of (**a**) 10 µM furanone treatment vs. control; (**b**) 10 µM VPL300 treatment vs. control; and (**c**) 100 µM VPL300 treatment vs. control. Left and right panels indicate KEGG categories that were activated and suppressed, respectively.

A gene set enrichment analysis was then conducted to annotate the DEGs using the Kyoto Encyclopedia of Genes and Genomes (KEGG), where the significance threshold of the *q* value was defined as <0.05. The positive control (10 µM of furanone treatment) revealed 24 (highest number) KEGG categories that were significantly enriched with DEGs, while the VPL300 treatments at 10 µM and 100 µM revealed 19 and 8 KEGG categories, respectively, which were significantly enriched with DEGs (Table A1). Most of the enriched KEGG categories were related to metabolisms, such as the pyruvate metabolism, fatty acid biosynthesis and metabolism, glycolysis/gluconeogenesis, amino acid biosynthesis and metabolisms, etc. Nevertheless, the quorum sensing was also found to be enriched with DEGs in all the treatment groups compared to the control (Table A1). All the treatment groups suppressed the quorum sensing (Figure A1), where the quorum-sensing-related gene expression was found to be down-regulated. In addition, the flagellar assembly was suppressed upon treatment with 100 µM of VPL300 (Figure A1c), although the enrichment analysis was not statistically significant (*q* value of 0.068), but the DEGs that were annotated in this pathway were also examined. The quorum-sensing- and flagellar-assembly-related gene expressions that were significant are shown in Figure 7 and Figure 8, respectively.

## 4. Discussion

**Figure A2:**
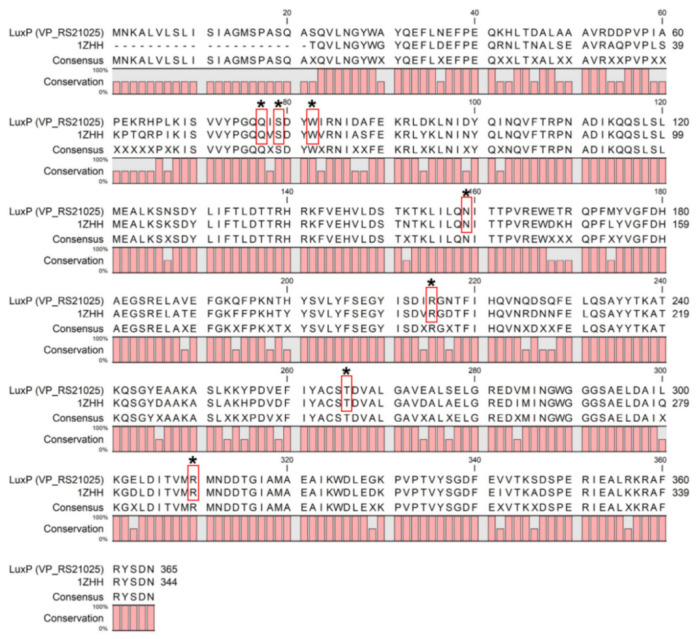
Sequence alignment of *V. harveyi* LuxP (PDB ID: 1ZHH) and *V. parahaemolyticus* LuxP (RS21025) showing an 84.59% sequence similarity. In addition, all 7 key amino acids that were involved in the binding of AI-2 were conserved in both sequences (highlighted in red box with *).

In this study, an in silico approach was applied to select DNA aptamer candidates against the LuxP periplasmic receptor protein. The advancements in computational biology and the emergence of bioinformatic tools have improved efforts to provide convenient platforms for high-throughput screening, as well as the precise prediction of biomolecular interactions based on the structural information of the molecules. This in silico approach to aptamer development has been reviewed extensively in the past [40,41,42]. In this study, due to the unavailability of a high-resolution crystal structure of the *Vibrio parahaemolyticus* LuxP receptor protein, the LuxP structure of the closest species, *V. harveyi*, was used for the molecular docking and dynamics simulation. A trustworthy and reliable protein model is crucial for computational analysis, especially molecular docking, and dynamics simulation [43]. A well-defined protein folding is critical for the prediction of ligand interactions. In order to prevent the decrement in the precision of the predicted protein–ligand interaction, the high-resolution crystal structure of the LuxP protein of the *Vibrio* species was used in this study. In addition, the LuxP receptor protein is known to undergo major conformational changes upon ligand interaction; thus, a well-defined overall fold of the protein is a prerequisite [43]. On a different note, the sequence alignment of the LuxP proteins used in this analysis showed an 84.59% similarity, and the key amino acids that are involved in the binding of the autoinducer-2 signaling molecule were conserved in both sequences (Figure A2)

Generally, the screening process of the in silico selection and characterization of aptamers involve the molecular docking of the oligonucleotides against the target. Various docking programs have been developed, and the commonly used programs include: AutoDock Tools [44], AutoDock Vina [45,46], HADDOCK [47,48], PatchDock [49], ZDOCK [50,51], etc. Computational docking enables the identification of oligonucleotides that bind to the target molecule and the listing of an initial set of oligonucleotide sequences with a high binding affinity against the specific target. The eight-mer oligonucleotide of VPL130 possesses the highest binding affinity based on the molecular docking analysis (Table 1). The analysis of the VPL130 binding pose (Figure 1b) identified 16 interacting amino acid residues, which included 6 of the key amino acids that are involved in the binding of the AI-2 signaling molecule (Figure 1a). The overlapping of the binding site explains the binding competition of the protein-binding aptamers with the endogenous ligand in many cases [52]. These aptamers are capable of the orthosteric regulation of the protein functions [52]. Thus, it was postulated that the LuxP-binding aptamer in this study could interfere with the bacterial quorum sensing. The interactions between LuxP and the selected oligonucleotides were analyzed using UCSF Chimera version 1.15, and a number of hydrogen bonding interactions were also identified. A higher number of interacting amino acids were observed (Figure 1), which enhance the stability of the binding. It has been reported that the aptamer length and modification affect its binding activity. For instance, a 36-mer aptamer showed a sub-nanomolar affinity to its target, but a 32-mer aptamer (four nucleotides shorter) was reported to lose its binding activity [53]. Thus, the four-mer oligonucleotides were extended to eight mers by combination in the current analysis. However, the shortfall of the current approach is that it omits the potential oligonucleotides that were not included in the core four-mer ones from the previous library. In spite of this, the four-mer oligonucleotides selected for combination to form eight-mer oligonucleotides consisted resulted in the highest binding affinity and were screened using the docking approach. In the further molecular dynamic simulation analysis, the intermolecular hydrogen bond analysis revealed that candidate VPL110 formed the highest average number of intermolecular hydrogen bonds (Figure 4). Hydrogen bonding is classified as a strong, non-covalent interaction that is particularly important in aptamer binding, in which the A, T, C, and G nucleobases are involved. Strong hydrogen bonds have been recorded with strengths up to 40 kcal/mol [54].

The MD simulation allowed for the further comprehension of the protein–oligonucleotide interactions and the conformational dynamics of the molecules [55]. The stability of the complexes that were assessed through the simulations using the GROMACS package showed minimal fluctuations in the protein backbone ranging from 1.5 to 2.5 Å (Figure 3a), indicating that the complex simulations were stable. However, the analysis of the hydrogen bond formation dynamics showed the formation and breaking of the hydrogen bonds during the course of the 50 ns simulation (Figure 4), compared to the predicted hydrogen bonds in the molecular docking analysis (Table 1), suggesting that the oligonucleotide sequences could be improved to form stable bonds with the active binding amino acid residues of the LuxP [40]. The fluctuation in the hydrogen bonds’ formation could also be due to the fluctuation in the conformational dynamics of the oligonucleotides and the amino acid residues. The molecular dynamics simulation revealed a detailed intermolecular interaction that is not comparable to the results of the rigid molecular docking. Nevertheless, all six complexes showed low binding free energies calculated using the MMPBSA approach, where VPL900 had the lowest binding free energy of −326.981 ± 38.291 kJ/mol. The molecular mechanics Poisson–Boltzmann surface area (MMPBSA) approach to the binding energy calculation is an efficient and reliable free energy calculation method that has been widely used [56,57,58]. It is crucial for binding energy calculations using the MMPBSA approach to obtain multiple different conformations or snapshots/timeframes over the course of the MD simulation in order to calculate the average energies [59]. Thus, 20 snapshots at time intervals of 500 ps from 30,000 to 40,000 ps of the simulations were obtained for the binding energy calculation, where the RMSDs of the complexes were stable (Figure 3). However, a longer MD simulation and higher number of snapshots used in the MMPBSA method for the binding energy calculation were expected to obtain a more reliable free energy calculation in this study.

Following this, the thermodynamics of the LuxP–aptamer interactions in the solution were determined using an isothermal titration calorimeter. Through the sensing of minute heat changes in the sample cell indicated by the calorimeter in a single experiment, this quantitative technique was able to provide multiple thermodynamic parameters of the biomolecular interactions. Prior to ITC, the selected eight-mer oligonucleotides were modified to avoid erroneous folding. The candidates were elongated at the 5′ end with a GGCC sequence and its complement at the 3′ end, defining a full sequence of the aptamer as 5′-GGCC-(8-mer oligos)-GGCC-3′, where the complement 5′ and 3′ ends form a rigid double helix structure. A similar modification using the TTAATT complement sequence at 5′ and 3′ ends was reported in a previous study that designed the cytochrome p450-binding aptamers through a computational approach [60]. The modification stabilized the aptamer structure, in which the self-complement 3′ and 5′ ends led to the formation of a hairpin structure. Out of the six aptamer candidates used in this study, only the VPL300 and VPL130 aptamers had a significant binding affinity against LuxP, where 0.2 and 0.5 micromolar of dissociation constants were recorded, respectively. The remaining four aptamer candidates showed a lower binding affinity that was not significant, where the dissociation constants recorded were higher than 1 micromolar. Nevertheless, an aptamer can exert a higher binding affinity at the nanomolar to sub-nanomolar level against the target, according to previous reports [53,61,62]. Interestingly, negative enthalpy changes (ΔH) and entropy changes (ΔS) were recorded for both of the aptamer candidates (Table 3), indicating that the Gibbs free energy may be positive or negative, depending on the temperature. Given the Gibbs free energy, ΔG is defined by the Gibbs equation: ΔG = ΔH − TΔS, where T is the temperature (Kelvin). Thus, reactions with a negative ΔH and positive ΔS are always spontaneous (negative Gibbs free energy). In this study, the negative Gibbs free energy indicates that the interactions were spontaneous at a low temperature for both the VPL300 and VPL130 aptamers, given that the ΔS results of both candidates were recorded to be negative (Table 3). The decreased ΔS could be related to the decreased conformational entropy of the LuxP in the aptamer-bound state. A previous study of the conformational dynamics and thermodynamics of protein–ligand binding elucidated the effects of protein conformational fluctuations on both the affinity and specificity of ligand binding [63]. Conformational changes in the aptamers and proteins upon their interactions have also been described previously [52]. Although structural comparisons revealed that the protein conformations were not significantly influenced by aptamer binding, in most cases, but the LuxP receptor protein is known to undergo major conformational changes upon ligand binding [64,65,66,67,68]. The superimposition of the apo- and holo-forms of the LuxP, which signifies the changes in the LuxP conformation upon AI-2 binding, has been well-documented [64,67]. The ordering or structuring of the interacting proteins upon binding are known to result in decreased conformational entropy [69]; thus, it is understandable that we observed this entropic penalty in the aptamer–LuxP binding in the current study.

Furthermore, the VPL300 aptamer was selected to evaluate its bioactivity in vitro, where the *V. parahaemolyticus* culture was treated with low (10 µM) and high (100 µM) doses of the aptamer. A positive control was included, in which the culture was treated with 10 µM of furanone, a known anti-quorum-sensing compound [70,71,72]. The comparative transcriptomic analysis revealed more than three thousand differentially expressed genes (DEGs) that were significant in *V. parahaemolyticus* upon the 10 µM furanone treatment. These DEGs were annotated in a wide range of metabolic KEGG categories (Figure A1a). This stands in contrast to the aptamer treatments where lower numbers of DEGs were identified, indicating that the aptamer treatments were less potent against the bacterial metabolisms. Nevertheless, the current analysis emphasizes the anti-quorum-sensing (QS) properties of the treatments, which aimed to suppress the virulence of *V. parahaemolyticus*. From the gene set enrichment analysis, the QS was found to be suppressed in all the treatments (Figure A1). The gene expression level of the QS master regulator at a high cell density, OpaR, was found to be significantly downregulated in the furanone treatment (Figure 7), but the suppression was not significant in the aptamer treatments. The role of OpaR in regulating QS has been frequently reported [73,74,75]. It is involved in biofilm formation, swarming, motility, and type-III and -VI secretion systems in *V. parahaemolyticus* [76,77]. Although the suppression of OpaR in the aptamer treatment was not significant, a number of polar flagellum genes were found to be significantly downregulated upon the 100 µM VPL300 treatment (Figure 8). Flagellum is a complex organelle that is crucial for bacterial mobility. It is also a potential virulence factor, where the flagellum act as an adhesin to mediate the adherence of the bacterial cells to the host [78]. A non-motile *Vibrio* mutant was reported to be defective with respect to adhesion and invasion in both in vitro and in vivo experiments [79,80,81,82]. Recently, the OpaR was reported to be a repressor of the polar flagellum genes in *V. parahaemolyticus* [83]. It was shown to bind to the promoter-proximal DNA regions within the polar flagellum gene loci to repress the polar flagellum gene transcription. Nevertheless, both the OpaR and polar flagellum genes were suppressed upon furanone treatment. Although the anti-QS effect of furanone was potent, the application of this halogenated furanone as a therapeutic agent was limited due to its potential toxicity and mutagenicity [84,85].

## 5. Conclusions

Aptamers are non-toxic organic molecules. They have clinical advantages as therapeutic agents. The current analysis demonstrated the potential of VPL300, designed using computational approach, in targeting the LuxP receptor protein and suppressing the QS and virulence in *V. parahaemolyticus* and, in particular, the expression of the polar flagellum genes that are crucial for bacterial motility. On the other hand, aptamers can be modified for improved bioactivities. Thus, the further modification of the VPL300 aptamer could be performed to increase its binding affinity against the LuxP receptor protein so as to enhance its bioactivities for the subsequent clinical development of this therapeutic aptamer against *Vibrio* infection in aquaculture.

## Figures and Tables

**Table 1:** Selected 8-mer oligonucleotides from the molecular docking analysis with the lowest binding energies, ranging between −7.5 and −9.2 kcal/mol. Important hydrogen bonds between the oligonucleotides and the amino acids are highlighted in bold. These amino acids were among the key residues forming the binding domain of the LuxP receptor protein that binds the autoinducer 2 signaling molecule.

Oligonucleotides		Affinity (kcal/mol)	Amino Acids Involved in Hydrogen Bonding
VPL300	ATAA GTGT	−7.5	Asn159; Ser293; Ala294; **Arg310**; **Arg215**; Ile211
VPL700	ATGG GTGG	−7.7	Gln116; Thr162; Ile211; **Arg215**; Asp136; Ser207
VPL900	GTGG AGCA	−8.4	Lys238; Ser207; Asp267; Ser293; **Thr266**; Ser265; Ala239; Asn159; Thr138; Thr137; Asp136; Tyr28; **Trp82**; Ala294
VPL110	AGCA TTAC	−8.1	Thr138; Ser207; **Asn159**; **Arg215**; **Thr266**; **Gln77**; Ser79; Pro109; Asp136; Tyr81
VPL120	TTAC AGCA	−8.0	Ile211; Tyr210; Asp136; Pro109; Thr138; Thr107; Thr162; **Ser79**; **Asn159**; **Arg310**
VPL130	TTAC AAGT	−9.2	**Trp82**; Thr134; His140; Ile211; **Arg215**; Thr137; **Arg310**; **Thr266**; Ala294; Arg84; Ser293; Asn312; **Ser79**; **Gln77**; Gln76; Gln116

**Table 2:** Summary of the binding energy based on the 50 ns molecular dynamics simulation, calculated using MMPBSA approach.

Oligonucleotides		MMPBSA Binding Energy (kJ/mol)
VPL300	ATAA GTGT	−251.615 ± 44.305
VPL700	ATGG GTGG	−265.427 ± 24.302
VPL900	GTGG AGCA	−326.981 ± 38.291
VPL110	AGCA TTAC	−260.524 ± 21.035
VPL120	TTAC AGCA	−301.524 ± 24.673
VPL130	TTAC AAGT	−293.980 ± 25.769

**Table 3:** Thermodynamic parameters of the interactions between LuxP and aptamer candidates.

Aptamer Candidates	K_d_ (M)	Stoichiometry Value, n	ΔH (kJ/mol)	Calculated ΔS (kJ/mol.K)	Calculated ΔG (kJ/mol)
VPL300	2.11 × 10^−7^ ± 2.03 × 10^−7^	0.928 ± 0.083	−84.37 ± 11.89	−0.1552	−38.12
VPL130	5.64 × 10^−7^ ± 5.29 × 10^−7^	0.746 ± 0.107	−51.91 ± 9.24	−0.05449	−35.67

## Data Availability

Not applicable.

## Supplementary Materials

The following supporting information can be downloaded at: https://www.mdpi.com/article/10.3390/biology11111600/s1, Figure S1: Purified LuxP protein in the elution fractions documented using 1D SDS-PAGE. The molecular weight of the purified LuxP protein is approximately 41 kDa; Figure S2: Alignment of the peptide sequences to the reference LuxP protein showed a 76.4% sequence coverage; Table S1: Peptide sequencing of the purified recombinant protein matched with the autoinducer 2-binding periplasmic protein LuxP of *V. parahaemolyticus*.

## Appendix A

**Table A1:** KEGG pathways enriched with differentially expressed genes by the gene set enrichment analysis (*q* value < 0.05).

KEGG	Set Size	Enrichment Score	NES	*p* Value	*q* Value
10 µM furanone treatment/control					
Oxidative phosphorylation	45	−0.73	−2.56	1.00 × 10^−10^	2.74 × 10^−9^
Butanoate metabolism	32	−0.84	−2.80	1.00 × 10^−10^	2.74 × 10^−9^
Two-component system	162	−0.46	−2.01	5.03 × 10^−8^	9.17 × 10^−7^
Citrate cycle (TCA cycle)	23	−0.78	−2.36	3.86 × 10^−7^	5.28 × 10^−6^
Valine, leucine and isoleucine degradation	29	−0.72	−2.31	1.31 × 10^−6^	1.43 × 10^−5^
ABC transporters	141	−0.44	−1.91	2.27 × 10^−6^	2.07 × 10^−5^
Quorum sensing	79	−0.52	−2.04	4.49 × 10^−6^	3.48 × 10^−5^
Microbial metabolism in diverse environments	207	−0.39	−1.77	5.08 × 10^−6^	3.48 × 10^−5^
Pyruvate metabolism	54	−0.58	−2.10	9.90 × 10^−6^	6.02 × 10^−5^
Biosynthesis of secondary metabolites	328	−0.34	−1.63	1.22 × 10^−5^	6.69 × 10^−5^
Carbon metabolism	106	−0.46	−1.90	2.06 × 10^−5^	1.02 × 10^−4^
Fatty acid metabolism	34	−0.62	−2.04	9.46 × 10^−5^	4.31 × 10^−4^
Propanoate metabolism	32	−0.62	−2.06	1.62 × 10^−4^	6.82 × 10^−4^
Fatty acid biosynthesis	25	−0.65	−2.01	2.78 × 10^−4^	1.09 × 10^−3^
Glutathione metabolism	18	0.70	1.87	1.08 × 10^−3^	3.95 × 10^−3^
D-Amino acid metabolism	16	−0.67	−1.86	1.60 × 10^−3^	5.47 × 10^−3^
Glycolysis/gluconeogenesis	35	−0.54	−1.81	2.03 × 10^−3^	6.55 × 10^−3^
Alanine, aspartate, and glutamate metabolism	33	−0.53	−1.76	4.42 × 10^−3^	0.01
Biotin metabolism	17	−0.64	−1.81	7.21 × 10^−3^	0.02
Methane metabolism	32	−0.52	−1.72	8.33 × 10^−3^	0.02
Tyrosine metabolism	13	−0.66	−1.74	8.89 × 10^−3^	0.02
Starch and sucrose metabolism	26	−0.54	−1.69	9.55 × 10^−3^	0.02
Fatty acid degradation	16	−0.61	−1.69	0.01	0.03
Arginine and proline metabolism	22	−0.56	−1.67	0.01	0.03
**10 µM VPL300 treatment/control**					
Biosynthesis of secondary metabolites	328	−0.51	−1.98	1.00 × 10^−10^	5.58 × 10^−9^
Biosynthesis of amino acids	119	−0.62	−2.16	3.11 × 10^−9^	7.65 × 10^−8^
ABC transporters	141	−0.59	−2.11	4.11 × 10^−9^	7.65 × 10^−8^
Valine, leucine and isoleucine degradation	29	−0.83	−2.31	8.29 × 10^−9^	1.16 × 10^−7^
Arginine biosynthesis	14	−0.87	−2.07	3.42 × 10^−6^	3.81 × 10^−5^
Histidine metabolism	14	−0.86	−2.04	8.33 × 10^−6^	7.75 × 10^−5^
Propanoate metabolism	32	−0.72	−2.07	1.84 × 10^−5^	1.29 × 10^−4^
Quorum sensing	79	−0.58	−1.92	1.86 × 10^−5^	1.29 × 10^−4^
beta-Lactam resistance	27	−0.70	−1.94	3.67 × 10^−4^	2.27 × 10^−3^
D-Amino acid metabolism	16	−0.78	−1.90	4.14 × 10^−4^	2.31 × 10^−3^
2-Oxocarboxylic acid metabolism	30	−0.67	−1.89	4.86 × 10^−4^	2.46 × 10^−3^
Glycine, serine, and threonine metabolism	40	−0.62	−1.83	6.14 × 10^−4^	2.86 × 10^−3^
Biotin metabolism	17	−0.74	−1.83	1.42 × 10^−3^	6.08 × 10^−3^
Purine metabolism	63	−0.53	−1.69	1.83 × 10^−3^	7.29 × 10^−3^
Fatty acid biosynthesis	25	−0.67	−1.83	2.24 × 10^−3^	8.35 × 10^−3^
Alanine, aspartate, and glutamate metabolism	33	−0.63	−1.83	2.58 × 10^−3^	9.01 × 10^−3^
Microbial metabolism in diverse environments	207	−0.38	−1.45	4.81 × 10^−3^	0.02
Tyrosine metabolism	13	−0.75	−1.74	7.30 × 10^−3^	0.02
Phenylalanine, tyrosine, and tryptophan biosynthesis	23	−0.62	−1.65	0.01	0.04
**100 µM VPL300 treatment/control**					
Ribosome	55	0.70	2.53	2.51 × 10^−10^	1.59 × 10^−8^
ABC transporters	141	−0.53	−2.19	7.50 × 10^−10^	2.37 × 10^−8^
Biosynthesis of secondary metabolites	328	−0.38	−1.72	2.67 × 10^−6^	5.63 × 10^−5^
Quorum sensing	79	−0.55	−2.05	5.37 × 10^−6^	8.48 × 10^−5^
Histidine metabolism	14	−0.81	−2.07	4.61 × 10^−5^	5.53 × 10^−4^
Biosynthesis of amino acids	119	−0.45	−1.82	5.26 × 10^−5^	5.53 × 10^−4^
Valine, leucine, and isoleucine degradation	29	−0.64	−1.97	1.70 × 10^−4^	1.53 × 10^−3^
Arginine biosynthesis	14	−0.70	−1.78	5.47 × 10^−3^	0.04
