# Supplementary material for: Computationally Designed Anti-LuxP DNA Aptamer Suppressed Flagellar Assembly- and Quorum Sensing-Related Gene Expression in Vibrio parahaemolyticus
Source: Biology (Basel). 2022 Nov 1;11(11):1600. doi: 10.3390/biology11111600 (PMC9687752; doi:10.3390/biology11111600)
Supplement: Supplementary file 1 [file biology-11-01600-s001.zip › Supplementary Table S1.pdf]

Table *s*1. Peptide sequencing of the purified recombinant protein matched to autoinducer 2-binding periplasmic protein LuxP of *Vibrio parahaemolyticus*.

Protein match	Peptide sequences
Autoinducer 2-binding periplasmic protein LuxP OS=Vibrio parahaemolyticus 901128 OX=1288792 GN=H334_14485 PE=4 SV=1	ATKQSGYEAAK
	SNSDYLIFTLDTTR
	QQSLSLMEALK
	QPFMYVGFDHAEGSR
	NTHYSVLYFSEGYISDIR
	NIDAFEKR
	MNDDTGIAMAEAIK
	ISVVYPGQQISDYWIR
	LNIDYQINQVFTRPNADIK
	LILQNITTPVREWETR
	LILQNITTPVR
	KYPDVEFIYACSTDVALGAVEALSELGR
	KFVEHVLDSTK
	HLTDALAAAVR
	GNTFIHQVNQDSQFELQSAYYTK
	GELDITVMRMNDDTGIAMAEAIK
	GELDITVMR
	FVEHVLDSTK
	EWETRQPFMYVGFDHAEGSR
	ELAVEFGKQFPK
	ELAVEFGK
	EDVMINGWGGGSAELDAILK
	TKLILQNITTPVR
	WDLEGKPVPTVYSGDFEVVTK
