# Supplementary figures and images for: Computationally Designed Anti-LuxP DNA Aptamer Suppressed Flagellar Assembly- and Quorum Sensing-Related Gene Expression in Vibrio parahaemolyticus
Source: Biology (Basel). 2022 Nov 1;11(11):1600. doi: 10.3390/biology11111600 (PMC9687752; doi:10.3390/biology11111600)

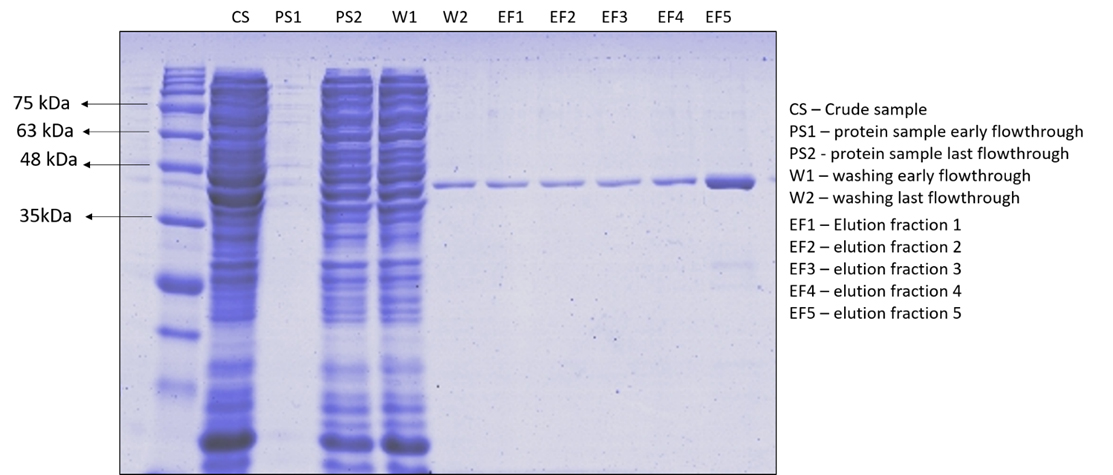

Supplement: Supplementary file 1 [file biology-11-01600-s001.zip › Supplementary Figure S1.jpg]

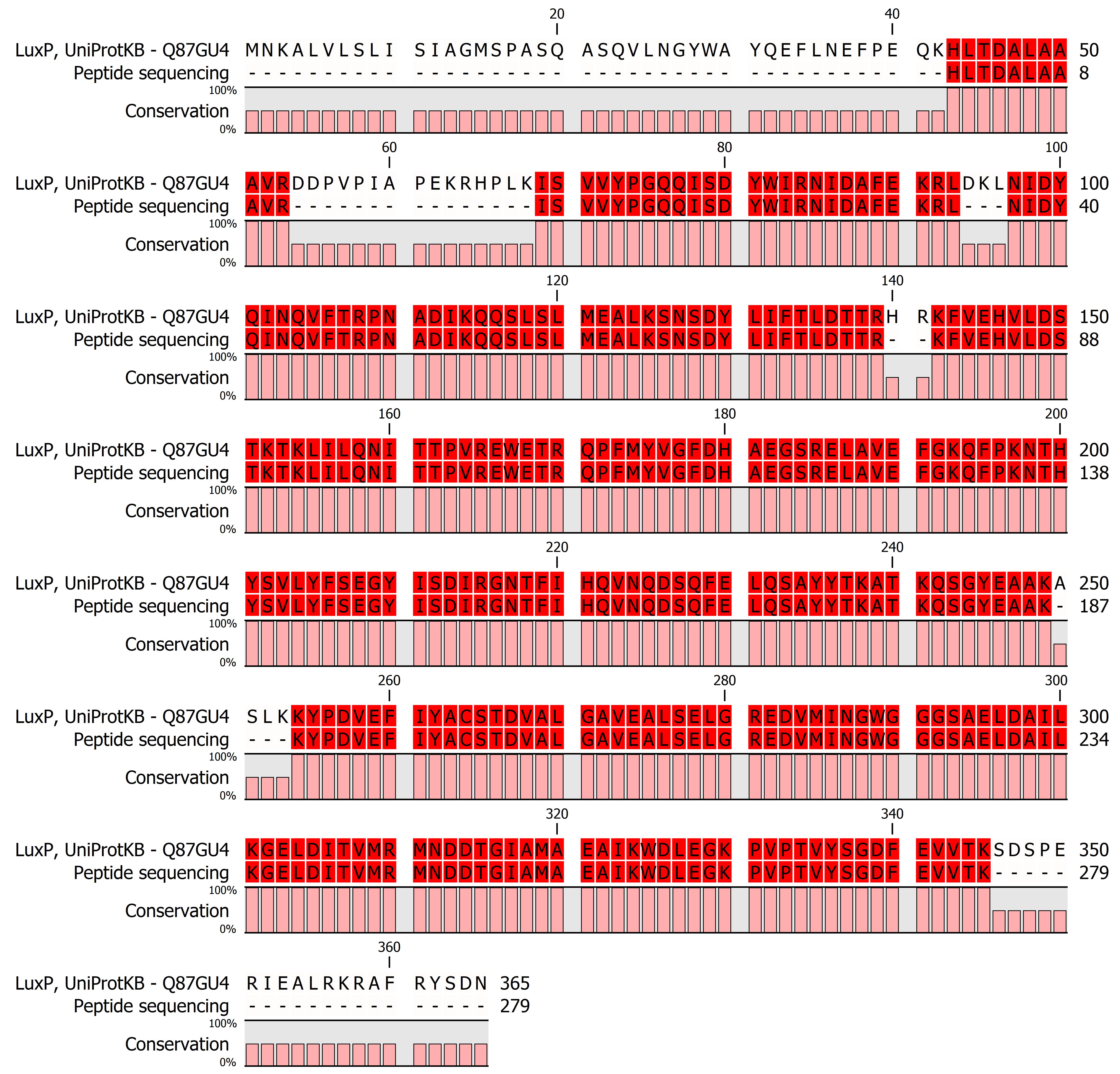

Supplement: Supplementary file 1 [file biology-11-01600-s001.zip › Supplementary Figure S2.jpg]
